# Molecular Epidemiology of Methicillin-Susceptible and Methicillin-Resistant *Staphylococcus aureus* in Wild, Captive and Laboratory Rats: Effect of Habitat on the Nasal *S. aureus* Population

**DOI:** 10.3390/toxins12020080

**Published:** 2020-01-24

**Authors:** Dina Raafat, Daniel M. Mrochen, Fawaz Al’Sholui, Elisa Heuser, René Ryll, Kathleen R. Pritchett-Corning, Jens Jacob, Bernd Walther, Franz-Rainer Matuschka, Dania Richter, Uta Westerhüs, Jiri Pikula, Jens van den Brandt, Werner Nicklas, Stefan Monecke, Birgit Strommenger, Sarah van Alen, Karsten Becker, Rainer G. Ulrich, Silva Holtfreter

**Affiliations:** 1 Department of Immunology, University Medicine Greifswald, 17475 Greifswald, Germany; dina.raafat@med.uni-greifswald.de (D.R.); mrochend@uni-greifswald.de (D.M.M.); Fawaz.AlSholui@med.uni-greifswald.de (F.A.); 2 Department of Microbiology and Immunology, Faculty of Pharmacy, Alexandria University, Alexandria 21521, Egypt; 3 Friedrich-Loeffler-Institute, Federal Research Institute for Animal Health, Institute of Novel and Emerging Infectious Diseases, 17493 Greifswald-Insel Riems, Germany; Elisa.Heuser@fli.de (E.H.); ryllrene@web.de (R.R.); rainer.ulrich@fli.de (R.G.U.); 4 Research and Professional Services, Charles River Laboratories, Wilmington, MA 01887, USA; pritchettcorning@fas.harvard.edu; 5 Julius Kühn-Institute, Federal Research Centre for Cultivated Plants, Institute for Plant Protection in Horticulture and Forestry, Vertebrate Research, 48161 Münster, Germany; jens.jacob@julius-kuehn.de (J.J.); bernd.walther@julius-kuehn.de (B.W.); 6 Outpatient Clinic, University of Potsdam, 14469 Potsdam, Germany; matuschka@frm-bioscience.de; 7 Institute of Geoecology, Landscape Ecology & Environmental Systems Analysis, Technische Universität Braunschweig, 38106 Braunschweig, Germany; dania.richter@tu-braunschweig.de; 8 von Opel Hessische Zoostiftung, 61476 Kronberg im Taunus, Germany; uta.westerhues@opel-zoo.de; 9 Department of Ecology and Diseases of Game, Fish and Bees, University of Veterinary and Pharmaceutical Sciences Brno, 61242 Brno, Czech Republic; pikulaj@vfu.cz; 10 CEITEC—Central European Institute of Technology, University of Veterinary and Pharmaceutical Sciences Brno, 61242 Brno, Czech Republic; 11 Central Core & Research Facility of Laboratory Animals, University Medicine Greifswald, 17475 Greifswald, Germany; brandtj@uni-greifswald.de; 12 German Cancer Research Center (DKFZ), Microbiological Diagnostics, 69120 Heidelberg, Germany; w.nicklas@outlook.de; 13 Leibniz Institute of Photonic Technology (IPHT), 07745 Jena, Germany; monecke@rocketmail.com; 14 Institute for Medical Microbiology and Hygiene, Technical University of Dresden, 01307 Dresden, Germany; 15 National Reference Centre for Staphylococci and Enterococci, Robert-Koch-Institute, Wernigerode Branch, 38855 Wernigerode, Germany; Strommengerb@rki.de; 16 Institute of Medical Microbiology, University Hospital Münster, 48149 Münster, Germany; sarah.vanalen@grunenthal.com (S.v.A.); karsten.becker@med.uni-greifswald.de (K.B.); 17 Friedrich Loeffler-Institute of Medical Microbiology, University Medicine Greifswald, 17475 Greifswald, Germany; 18 German Center for Infection Research (DZIF), Partner Site Hamburg-Lübeck-Borstel-Insel Riems, 17493 Greifswald-Insel Riems, Germany

**Keywords:** *Staphylococcus aureus*, rat, clonal complex, host adaptation, livestock, laboratory, coagulation, immune evasion cluster, habitat, epidemiology

## Abstract

Rats are a reservoir of human- and livestock-associated methicillin-resistant *Staphylococcus aureus* (MRSA). However, the composition of the natural *S. aureus* population in wild and laboratory rats is largely unknown. Here, 144 nasal *S. aureus* isolates from free-living wild rats, captive wild rats and laboratory rats were genotyped and profiled for antibiotic resistances and human-specific virulence genes. The nasal *S. aureus* carriage rate was higher among wild rats (23.4%) than laboratory rats (12.3%). Free-living wild rats were primarily colonized with isolates of clonal complex (CC) 49 and CC130 and maintained these strains even in husbandry. Moreover, upon livestock contact, CC398 isolates were acquired. In contrast, laboratory rats were colonized with many different *S. aureus* lineages—many of which are commonly found in humans. Five captive wild rats were colonized with CC398-MRSA. Moreover, a single CC30-MRSA and two CC130-MRSA were detected in free-living or captive wild rats. Rat-derived *S. aureus* isolates rarely harbored the phage-carried immune evasion gene cluster or superantigen genes, suggesting long-term adaptation to their host. Taken together, our study revealed a natural *S. aureus* population in wild rats, as well as a colonization pressure on wild and laboratory rats by exposure to livestock- and human-associated *S. aureus*, respectively.

## 1. Introduction

*Staphylococcus aureus* (*S. aureus*) is a major opportunistic pathogen in human medicine and it is increasingly recognized as a zoonotic pathogen. The nasal cavity of approximately 20% of adult humans is persistently and asymptomatically colonized with *S. aureus* [1,2,3]. These bacteria can cause a wide variety of illnesses, ranging from skin and soft tissue infections (e.g., abscesses) to life-threatening diseases (e.g., endocarditis and sepsis) [4]. The emergence of multiresistant (methicillin-resistant *S. aureus*, MRSA; vancomycin-resistant *S. aureus*, VRSA) and highly virulent (community acquired MRSA, CA-MRSA) strains makes *S. aureus* a prominent example of the antibiotic resistance crisis and a major public health concern worldwide [5,6,7]. This medical predicament is especially heightened by the fact that to date, no *S. aureus* vaccine is available [8].

Besides humans, *S. aureus* is also able to colonize and infect numerous other species, including companion animals, livestock as well as wild animals [9,10,11,12,13]. Transmission of MRSA occurs primarily upon person-to-person contact, but MRSA can also spread between domestic animals and people [14,15]. The recent detection of MRSA in rats suggests that pest animals might be an additional reservoir for MRSA [16,17,18,19,20]. For instance, wild rats caught on farms were carrying typical livestock-associated (LA)-MRSA strains (e.g., clonal complex (CC) 398 and sequence type (ST) 97) [18,21], while urban rats carried the same MRSA lineages prevalent in the respective local human or animal population (e.g., USA300-ST8) [19]. The natural *S. aureus* population in rats, which is likely dominated by methicillin-susceptible *S. aureus* (MSSA), is largely unknown [21]. In view of the emerging zoonotic potential of certain *S. aureus* lineages [22,23,24], an effective “One Health” strategy against the spread of *S. aureus* and especially MRSA has to take these bidirectional transmissions between animals and humans into account [25,26].

The ability of *S. aureus* to colonize various host species is favored by its plastic genome, which encompasses the core genome (75%), the core variable genome (10%) and the accessory genome (15%). The core variable genome contains the information for surface proteins and transcription regulators, while the accessory genome consists mainly of mobile genetic elements (MGEs), and thus represents the most variable part of the genome [27,28,29]. Unsurprisingly, the accessory genome plays a major role in adaptation processes of *S. aureus* [30], allowing it to adapt to changing environments, such as different host species. Prominent examples include: (i) lack of antibiotic resistance genes and the highly human-specific immune evasion gene cluster (IEC) in animal isolates; (ii) lack of superantigen genes in murine *S. aureus* isolates; (iii) acquisition of variants of the von Willebrand factor-binding protein in ruminant and equine strains, carried by highly mobile pathogenicity islands (SaPIs); as well as (iv) a single nucleotide mutation in the *dltB* gene, a gene involved in lipoteichoic acid biosynthesis, which enabled a human strain to infect rabbits [10,11,31,32,33,34].

These host adaptation processes frequently restrict the host range that can be successfully colonized and infected by a certain *S. aureus* lineage. In consequence, certain CCs seem to have a restricted host spectrum, like ST5 in poultry or ST433 in pigs [35,36], while others, like CC8, CC22 and CC398, display an extended host spectrum and are therefore called Extended-Host-Spectrum Genotypes (EHSGs) [24,37,38].

Along with mice, rats are a common animal model for studying *S. aureus* pathogenicity and new intervention strategies [39,40,41]. While investigating the epidemiology of *S. aureus* in both wild and laboratory mice in previous studies, we have found distinct clonal lineages and hints for host adaptation [11,34,42]. Moreover, certain mouse- and vole-adapted *S. aureus* strains turned out to be suitable tools to improve murine infection models by allowing persistent intranasal colonization or strongly reducing the required inoculation dose in infection models [42,43]. Despite their importance in infection models, the prevalence of *S. aureus* in laboratory rats, and its mechanisms of host adaptation, has yet to be investigated.

The major aim of this study was to extend our knowledge of *S. aureus* (esp. MRSA) carriage in rats. This was achieved by collecting *S. aureus* strains from free-living and captive wild rats, as well as laboratory rats, followed by determining the population structure and antibiotic resistance profiles of these *S. aureus* isolates. Moreover, we looked for signs of host adaptation by screening for bacterial genes known to be involved in this process.

## 2. Results

### 2.1. Laboratory Rats and Wild Rats Are Colonized with S. aureus

This study aimed at determining the prevalence and molecular epidemiology of *S. aureus*, including MRSA, in free-living and captive wild rats, as well as laboratory rats. Therefore, 145 free-living wild rats captured between 2009 and 2017 in three German federal states as well as in the Czech Republic were analyzed for *S. aureus* nasal carriage (Tables S1 and S2). In addition, a total of 188 captive wild rats originating from three German federal states and held in laboratory housing or large enclosures for several years were investigated for *S. aureus* nasal carriage (Tables S3 and S4). Furthermore, 114 laboratory rats, raised as experimental or feeder animals, from four German federal states were included (Tables S3 and S5). Finally, *S. aureus* isolates from 52 laboratory rats held in Germany, USA, Japan and Canada were analyzed (Table S5).

Overall, the *S. aureus* carriage rate was similar among free-living wild rats (37/145; 25.5%) and captive wild rats (41/188; 21.8%), but lower among laboratory rats (14/114; 12.3%) (Table 1). However, within the different categories, the prevalence differed strongly according to the geographical location. In free-living wild rats, the highest prevalence was seen in the Moravian-Silesian Region (MSR, 62.1%), followed by the German states Mecklenburg-Western Pomerania (MV, 22.2%), and then North Rhine-Westphalia (NRW, 17.3%) (Table 1 and Table S6). The lowest prevalence was observed in pest animals from a zoo in Baden-Württemberg (BW, 5.9%). This divergence was even more pronounced among captive wild rats, where breeding procedures and hygiene measures can promote or prevent the spread of *S. aureus*, respectively. For instance, *S. aureus* prevalence was very high (77.1%) in captive wild rats in Berlin (BE) (Table 1, Tables S4 and S7). In contrast, one wild rat population caught at a farm and afterwards held in husbandry in NRW was completely *S. aureus*-free. Moreover, we observed *S. aureus*-positive (e.g., Neufels, Tilbury) and -negative (e.g., BSR, WPHR) rat strain populations in the same husbandry in Brandenburg (BB) (Tables S4 and S7). This suggests that the transmission between strains is effectively blocked by hygiene measures. Colonization rates among laboratory rats were also diverse and ranged from 0.0% (BW, NRW) to 33.3% (MV). 

### 2.2. Wild Rats, Rats with Contact with Livestock and Laboratory Rats Carry Different S. aureus Clonal Complexes

**Figure 1:**
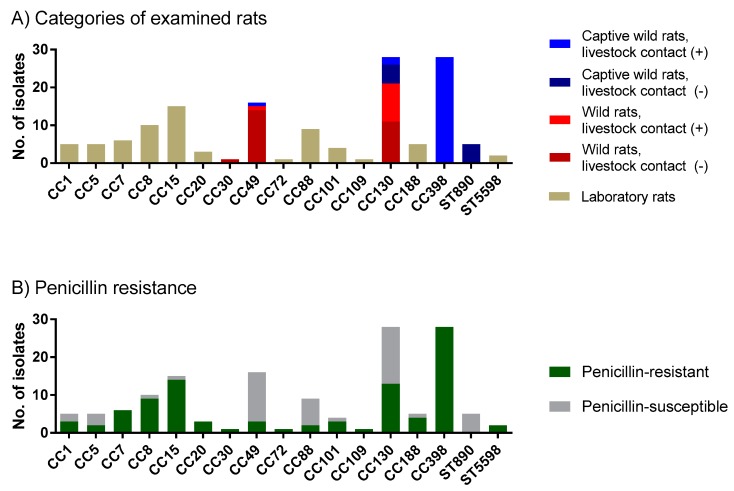
Wild rats and laboratory rats differ in their colonizing *S. aureus* population and the prevalence of penicillin-resistant *S. aureus*. *S. aureus* isolates were obtained from free-living as well as captive wild rats, in addition to laboratory rats. For a definition of the different rat categories, please refer to the legend of Table 1. In addition, 52 *S. aureus* strains isolated from laboratory rats from Germany, USA, Japan and Canada and characterized in this study were included in the analysis. The *S. aureus* population structure was resolved by *spa* typing; related *spa* types were grouped into CCs. (**A**) Isolates were clustered according to their origin (free-living wild rats, captive wild rats, laboratory rats). *S. aureus* isolates from free-living wild rats with direct contact with livestock or from captive wild rats whose ancestors have had contact with livestock are depicted in a lighter shade. (**B**) Prevalence of penicillin resistance in the different lineages. Minimum inhibitory concentration (MIC) values for penicillin were determined with the broth microdilution method.

The *S. aureus* population structure in our described rat cohorts was resolved by *spa* typing. Free-living wild rats were predominantly colonized with two *S. aureus* lineages: CC130 (N = 21) and CC49 (N = 15) (Figure 1A and Table S6). In addition, a single CC30 isolate was detected in a free-living wild rat caught in a German town. 

Interestingly, captive wild rats held in laboratories or large enclosures for several years were colonized either with the above described rat-related lineages CC130 (N = 7) and CC49 (N = 1), the wild mouse-related lineage ST890 (N = 5) or with the livestock-associated lineage CC398 (N = 28) (Figure 1A and Table S7) [11]. For instance, wild rats whose predecessors were captured on a pig farm in 2015 (strain Neufels) and kept under laboratory settings in two separate husbandries (BB and BE) were mostly (28/41; 68.3%) colonized with CC398 (*spa* type t011) (Tables S4 and S7). However, while the BB rats were colonized with CC398-MRSA (*mecA*-positive; see below), the BE rats carried CC398-MSSA. These data imply that contact with livestock is a significant risk factor for the acquisition of LA-*S. aureus* isolates. In contrast, the offspring of wild rats captured in a city were colonized with CC130-MSSA and ST890-MSSA. *S. aureus* lineages commonly found in humans were not detected in these animals. 

On the other hand, laboratory rats raised as experimental animals or feeder animals harbored a broad range of lineages (Figure 1A and Table S8). The most frequently detected lineage was CC15 (N = 15), followed by CC8 (N = 10), CC88 (N = 9), CC7 (N = 6), CC1 (N = 5), CC5 (N = 5), and CC188 (N = 5). Many of those are common in the human population, e.g., CC1, CC5, CC7, CC8, CC15, and CC20 [44]. In addition, we detected a novel multilocus sequence type (MLST) in this cohort in a laboratory rat from BW (MLST5598; *spa* type t2091). Notably, there was no overlap between the *S. aureus* populations in laboratory rats and wild rats (both free living and captive). 

**Figure 2:**
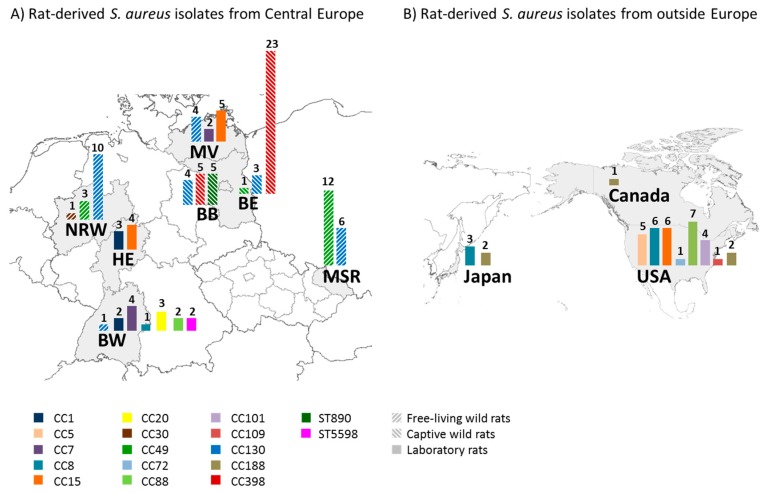
The *S. aureus* lineages CC49 and CC130 are widespread among wild rats, whereas CC8, CC15 and CC188 are widespread among laboratory rats. The graph illustrates the occurrence of *S. aureus* lineages (CC) in different federal states of Germany and the Czech Republic (**A**), as well as in Japan, Canada and the USA (**B**). For a definition of the different rat categories, please refer to the legend of Table 1. For captive wild rats, the location of the husbandry, rather than their capture location, is depicted. The different lineages are color coded; *S. aureus* isolates from free-living and captive wild rats are depicted in differentially-striped bars. *S. aureus* isolates from laboratory rats are shown in solid bars. The absolute number of isolates is depicted on top of the bars. Abbreviations: BB, Brandenburg; BE, Berlin; BW, Baden-Württemberg; HE, Hesse; MV, Mecklenburg-Western Pomerania; NRW, North Rhine-Westphalia; MSR, Moravian-Silesian Region.

To determine whether there are differences in the geographical distribution of the *S. aureus* lineages, all determined lineages were assigned to a map. For captive wild rats, the location of the husbandry, rather than their capture location, is depicted. Among wild rats (both free-living and captive), CC130 was most widely spread (Figure 2A). This lineage was detected in several rats from five German federal states (BB, BE, BW, MV, and NRW), as well as in the Czech Republic (Moravian-Silesian Region, MSR). Similarly, CC49 was detected in both free-living and captive wild rats originating from BE, NRW and the Czech Republic (MSR).

Among the laboratory rats, CC8, CC15 and CC188 were most widely spread, with CC8 being detected in rats from three continents (Figure 2A,B). CC1, CC7, and CC88 were observed in two locations, while the remaining lineages were detected only at a single location. The validity of these data is, however, restricted by the limited sampling size in some geographical locations (Table 1).

### 2.3. Penicillin Resistance Was Low in Wild Rats but High in Rats with Livestock Contact

We have previously reported that murine *S. aureus* isolates show a lower prevalence of penicillin resistance than human strains [34]. To extend these findings, we determined the MIC of penicillin for all rat-derived *S. aureus* isolates using the broth microdilution method. Overall, 95/144 (66.0%) isolates were penicillin-resistant, however the prevalence varied significantly between the groups (Chi-Squared test, *p* < 0.001). While the resistance rate was low in *S. aureus* isolates from free-living wild rats (15/37; 40.5%), it averaged 73.2% (30/41) in captive wild rats and 75.8% (50/66) in laboratory rats. The highest rate of penicillin resistance (96.3%; 26/27) was observed in rats with livestock contact (Table 1, Tables S6 and S7). This pattern is also reflected by varying rates of penicillin resistance in *S. aureus* lineages associated with either free-living wild rats (CC49, CC130), captive wild rats (CC130, ST890), laboratory rats (various CCs) and captive wild rats with previous livestock contact (CC398) (Figure 1A,B). 

### 2.4. MRSA Was Detected among Wild Rats, but Not among Laboratory Rats

In general, several wild rats were colonized with MRSA, while the laboratory rat population was free of MRSA (Table 2). In total, 6/188 (3.2%) captive wild rats and 2/145 (1.4%) free-living wild rats were colonized with MRSA. Five out of six MRSA-positive captive wild rats were colonized with CC398-MRSA. Interestingly, all affected rats were derived from the same rat strain (Neufels) in BB within the same year. These animals originated from black rats (*Rattus rattus*) caught at a livestock farm in Southern Germany and have been bred in BB since 2015. All five strains belonged to the same *spa*-type (t011), carried the methicillin-resistance gene *mecA* and showed an identical resistance pattern, suggesting the maintenance and spread of a single MRSA clone during captive breeding (Table 2).

Moreover, we detected two *mecC*-encoding CC130 isolates (*spa* type t843) in wild rats. One of the rats was caught in a zoo as pest rodent, while the other was a captive wild rat. In addition, a Norway rat positive for CC30-MRSA encoding the *mecA* gene was caught in a city. All three isolates were phenotypically resistant to cefoxitin, but sensitive to oxacillin, suggesting a heterogenous expression of the PBP2a protein. *mecB-* and *mecD*-positive *S. aureus* isolates were not detected in this study.

### 2.5. S. aureus Isolates from Rats Show Features of Host Adaptation

We have previously reported that murine *S. aureus* isolates from laboratory and wild mice and voles have adapted to their rodent host by eliminating human-specific phage-carried immune evasion (IEC) genes as well as MGE-carried SAg genes [11,34,45]. To test whether *S. aureus* isolates from rats show similar features of host adaptation, we compared the presence of IEC genes and the SAg gene repertoire with CC-matched human isolates. 

Sa3int phages harbor the human-specific IEC encoding staphylokinase (SAK), staphylococcal complement inhibitor (SCIN), chemotaxis inhibitory protein of *S. aureus* (CHIPS) as well as staphylococcal enterotoxins A or P (SEA, SEP) [46,47]. IEC-encoding prophages are prevalent in human *S. aureus* isolates, but frequently absent in animal-adapted strains, including murine and vole *S. aureus* isolates [11,34,44]. In line with this, only 1/37 (2.7%) isolates from free-living wild rats, none of the *S. aureus* isolates from captive wild rats (0/41) and 39/66 (59.1%) of laboratory rats harbored the phage-carried IEC genes *sak*, *chp* and/or *scn* (Tables S6–S8). A CC-wise comparison of the most prevalent rat *S. aureus* lineages with matched human strains revealed that IEC genes were significantly less frequent or even absent in the rat-derived CC8, CC49, CC88, and CC398 isolates (Table 3). 

SAg genes are prevalent in human *S. aureus* isolates (ca. 80%, not considering *selX* in the core genome) [44]. In contrast, only 1/37 (2.7%) isolates from free-living wild rats, none of the *S. aureus* isolates from captive wild rats (0/41) and 21/66 (31.8%) isolates of laboratory rats harbored SAg genes. In 12 of these 22 strains, these SAg genes were located on MGEs (Tables S6–S8). A CC-matched analysis revealed that MGE-carried SAg genes were significantly less frequent in rat CC8 and CC88 isolates than their human counterparts (Table 3). For instance, seven out of 10 human CC8 isolates harbored the plasmid-carried staphylococcal enterotoxin genes *sed/sej/ser*, the phage-carried *sea/sep*, and/or the SaPI-carried *seb*/*sek*/*seq (*Table 3 and Table S9*)*. In contrast, all ten rat CC8 isolates lacked SAg genes (Table S8). Similarly, 50% of human CC88 isolates were positive for SAg genes, while all nine rat CC88 isolates were negative (Tables S8 and S9). Taken together, *S. aureus* isolates from laboratory rats and wild rats rarely harbor phage-carried IEC genes and MGE-carried SAg genes, suggesting long-term adaptation to their hosts.

### 2.6. Both CC and Origin of S. aureus Determine Its Coagulation Behavior

**Figure 3:**
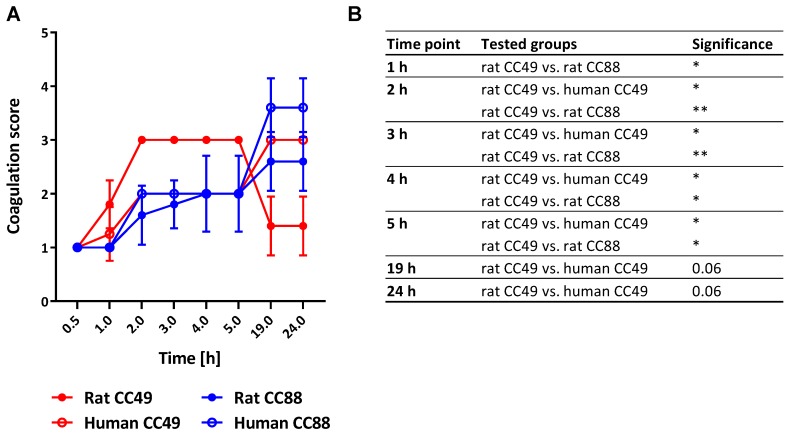
The *S. aureus* lineage CC49 originating from rats shows a different coagulation behavior in rat plasma than human CC49, human CC88 and rat CC88 strains. (**A**) The graph shows the coagulation of rat plasma by both human and rat CC49 and CC88 isolates (N = 4–5/group) over a time course of 24 h. The medium control did not induce any coagulation (data not shown). The graph depicts representative data from one of four consecutive experiments with concordant results. (**B**) Differences in the coagulation potential between the groups (rat CC49 vs. human CC49; rat CC88 vs. human CC88; human CC49 vs. human CC88, rat CC49 vs. rat CC88) were statistically evaluated per time point by a Kruskal-Wallis test with Dunn’s multiple comparisons test. The asterisks represent the adjusted *p* value: *, *p* value < 0.05; **, *p* value < 0.01.

The coagulation of the host’s plasma is a typical virulence trait of host-adapted *S. aureus* strains [31]. None of the tested strains was able to completely coagulate plasma obtained from Sprague Dawley rats (Figure 3). Nevertheless, the formation of small to large clots was observed, and after 24 h of incubation, human CC88 strains formed the biggest clots. Looking at the kinetics of coagulation, rat CC49 strains coagulated rat plasma faster than all other tested strains whose coagulation behavior was similar within the first 5 h. Moreover, the rat CC49 strains were the only ones able to reduce the coagulation score, i.e., disintegrate the already formed clots, over time.

## 3. Discussion

While wild rats are a known reservoir for MRSA, the natural *S. aureus* population in wild and laboratory rats has not been described in detail [18,19,21]. Here, we determined the prevalence of *S. aureus* including MRSA in free-living and captive wild rats, as well as laboratory rats (Table 1). We observed striking differences between these three major cohorts. Free-living wild rats as well as those captured and held in husbandry were mainly colonized with the *S. aureus* lineages CC49 and CC130. In contrast, laboratory rats were colonized with various lineages, mostly of human origin (Figure 1). Rats with contact with livestock were frequently colonized with the livestock-associated lineage CC398; some of the isolates were even methicillin-resistant (Figure 1, Table 2). *S. aureus* isolates from rats showed features of host adaptation on both the genetic as well as the phenotypic level (Table 3). These results underline the importance of rats as reservoir for MSSA as well as MRSA. 

### 3.1. Wild Rats Are Predominantly Colonized with The S. aureus Lineages CC49 and CC130 

One-quarter (25.5%) of the examined wild rats were positive for *S. aureus*. These *S. aureus*-positive rats were detected in different areas of Germany and the Czech Republic over a time period of eight years, suggesting that *S. aureus* colonization of rats is widely distributed. Hence, rats can be considered as a natural host of *S. aureus*. Compared to other species, e.g., humans, the diversity of the detected lineages is quite low, although this might be affected by the small sample size in some cohorts [44]. Interestingly, the two dominating lineages, CC130 and CC49, could also be found in wild mice and voles, as we have reported previously [11]. The lineage CC130 has already been isolated from a Norway rat [10] and shows a broad host spectrum, especially in wildlife. It has been detected in red foxes, hedgehogs, brown hares, wild boar, rabbits and magpies all over Europe and Tunisia [10,48,49,50]. In contrast to our study, most of the previously described CC130 isolates were MRSA, harboring the *mecC* locus. 

CC49 strains show a less diverse host spectrum and have mostly been isolated from wild animals such as bank voles, squirrels and wild boar in Central Europe and Spain [10,11,51,52,53]. Usually, these isolates show an MSSA phenotype, but MRSA (both *mecA* and *mecC* positive) were also found in pigs, horses, and humans [54,55,56]. This suggests that SCC*mec* elements are predominantly acquired and maintained in environments with significant selective pressure due to the use of antibiotics [57,58].

### 3.2. Rats with Contact with Livestock Frequently Carry CC398 

Captive wild rats whose predecessors were captured on a pig farm in 2015 (strain Neufels) and kept under laboratory settings in two separate husbandries (BB and BE) were mostly (90.3%) colonized with CC398-MSSA (Tables S4 and S7). However, while the BB rats were colonized with CC398-MRSA (*mecA*-positive), the BE rats carried CC398-MSSA. CC398-MRSA have been repeatedly reported from rats trapped on farms and even within inner city centres [18,19,21]. Since two of these studies focused only on MRSA, epidemiological data on CC398-MSSA are rather scarce.

CC398-MRSA are highly prevalent in German livestock, but CC398-MSSA have also been reported [59,60]. Thus, it can be assumed that the CC398 strains were acquired from livestock and spread within the rat population, as this type of acquisition and spread has already been described for other lineages and host species [30,35,61]. There are several possible scenarios: First, CC398-MRSA could have been transmitted from livestock to rats and subsequently lost the SCC*mec* element in BE husbandry. Second, CC398-MSSA could have been transferred from pigs to rats and the BB subpopulation subsequently acquired the SCC*mec* element. Third, both CC398-MSSA and -MRSA could have been transmitted to rats with the former being maintained in the BE and the latter in the BB rat population. The detection of a LA-*S. aureus* lineage in captive wild rat populations years to decades after acquisition of the ancestral animals suggests a stable colonization of rats and an effective transmission to their offspring, similar to what has been observed in laboratory mice [45]. Since CC398 belongs to the EHSG, it is still unclear whether this effective acquisition of LA-*S. aureus* strains holds true for all *S. aureus* lineages or just to certain ones. Nevertheless, we can conclude that wild rats can act as reservoir for LA-*S. aureus*, including LA-MRSA, making the containment of those pathogens even more challenging than previously assumed. 

### 3.3. Laboratory Rats Carry Various Lineages, Mostly of Human Origin

Laboratory rats carried a high diversity of *S. aureus* lineages—many of which are commonly found in humans, e.g., CC1, CC5, CC8, CC15 and CC101 [44]. Considering the close contact of laboratory rats with humans over many decades, it is only reasonable to assume that these strains have originally been transmitted from humans to rats. This might be supported by the fact that there is a significant overlap with the *S. aureus* population in laboratory mice [34], implying that humans could be the prime source of new *S. aureus* strains in laboratory animals. Transmission of *S. aureus* from humans to animals has been described several times [62]. However, it seems likely that not all human-adapted lineages are able to colonize rats. For instance, the most common human lineages (CC30 and CC45) are almost completely absent in the rat population and have also only rarely been detected in laboratory mice [34,44].

On the other hand, colonizing lineages such as CC88 and CC188 have already been described in other rodents and are rarely found in humans, suggesting that they might represent rodent-specific lineages [11,34]. Their origin, however, remains unclear. They might have originated from the human population and introduced into the rat colony by animal caretakers, with subsequent adaptation to their new rodent host over time. Alternatively, they might have already been present in the ancestry rat population at the time when commercialized breeding was established. Nevertheless, the low prevalence of phage-carried IEC genes and MGE-carried SAg genes in all rat-derived *S. aureus* lineages suggests a long-term colonization of these hosts. 

### 3.4. Captive Wild Rats Maintain Their S. aureus Population

Three of the analyzed rat husbandries (located in the German federal states BB, BE, and NRW) harbored wild rats that have been maintained in captivity for several years. Two of these husbandries were *S. aureus*-positive (19.4% in BB, 77.1% in BE), while in the NRW cohort, *S. aureus* was completely absent. Within the same husbandry in BB both *S. aureus*-positive (e.g., Neufels, Tilbury) and -negative (e.g., BSR, WPHR) rat strains were detected (Table S4). This suggests that a transmission between different rat strains was effectively prevented by the implemented hygiene measures. This finding is supported by similar observations in laboratory mice [45]. 

In addition, the captive wild rats are of interest, because they allow some conclusions about the stability of the *S. aureus* population after adaptation to a new environment with no further contact with wildlife, but increased contact with humans. The *S. aureus*-positive cohorts of captive wild rats (located in BB, and BE) were still colonized with *S. aureus* lineages common in wild rats (CC49, CC130) and livestock (CC398), while typical human *S. aureus* lineages, which were common in laboratory rats (see above), were not detected, even though they included animals crossed with laboratory rats (Table S5). This may be explained by a high stability of the *S. aureus* population in the rat cohort [63]. The absence of typical human *S. aureus* lineages suggests that several factors, including the existing microbiome [64,65,66], the limited contact of caretakers to animals, as well as high hygienic standards (including wearing coats and gloves) successfully prevent the introduction of human-adapted *S. aureus* strains. Moreover, the rat-adapted *S. aureus* population may be able to outcompete introduced human-adapted *S. aureus* strains. Indeed, we have previously observed that the *S. aureus* population in laboratory mice is stable over years, and that these bacteria are very effectively transmitted to their offspring [45]. Whether this holds true for rats as well needs to be further investigated.

### 3.5. S. aureus Isolates from Rats Likely Adapt to Their Host by Eliminating MGEs Carrying Human-Specific Virulence Factors 

A comparison of rat and CC-matched human *S. aureus* isolates showed that the rat strains are probably adapted to their host on both genetic and phenotypic levels. The observed scarcity of the phage-carried IEC genes, especially among *S. aureus* isolates from wild rats, indicates a strong selection against these genetic elements. Several phage-encoded immune evasion factors show no or little activity on rat cells [67,68,69,70]. For example, both CHIPS and SCIN efficiently block complement activation in humans but show no activity in mice and rats [67,70]. In contrast, SAK shows thrombolytic activity also in rats [71]. In addition, the Sa3int phage integrates into and thereby inactivates a known staphylococcal virulence gene, the sphingomyelinase hemolysin beta (*hlb*) gene. Hlb lyses erythrocytes, lymphocytes and keratinocytes and contributes to skin colonization in mouse models [72]. Thus, eliminating the Sa3int phage and thereby restoring the *hlb* gene could be advantageous for colonizing rats. In line with this, we have previously observed that this prophage is rare in *S. aureus* populations from laboratory and wild mice [11,34,45]. Indeed, the lack of the phage-carried IEC genes is a well-known marker for *S. aureus* isolates of animal origin [46,73,74]. 

Another genetic correlate of host adaptation was the absence of MGE-carried SAg genes in the lineages CC8 and CC88. This confirms previous observations in wild and laboratory mice, where *S. aureus* isolates also lacked MGE-carried SAg genes. *S. aureus* SAgs activate human T cells in the picomolar concentration range in vitro but show a 100–1000-fold reduced activity on rat T cells and a 10–100-fold reduced activity on murine T cells [69,75,76]. Elimination of SAg genes in *S. aureus* isolates from rats, whenever their genetic location permits this, underlines their expendability in rats.

### 3.6. Rat-derived CC49 Isolates Show Enhanced Procoagulatory Activity on Rat Plasma

Blood coagulation is a major virulence trait of *S. aureus*, and some pro- or anticoagulatory factors are known to be host specific [31]. Therefore, we compared the pro-coagulatory activity of CC49 and CC88 isolates from rats with their matched human counterparts. Rat CC49 isolates coagulated rat plasma faster than matched human CC49 isolates. In contrast, we observed no difference in our test system for the CC88 isolates. In line with our data, Viana et al. reported that ruminant-derived strains, but not human isolates, had the capacity to stimulate clotting of ruminant plasma due to a unique MGE-encoded paralogue of the von Willebrand factor binding protein [33]. The molecular basis for the observed differences in our rat-derived *S. aureus* isolates will be clarified by whole-genome sequencing in future studies.

### 3.7. Rats Carrying Human-Derived or LA-MRSA Present a Human Health Risk 

Considering the growing public health relevance of LA-MRSA, i.e., CC398-MRSA, in human health and livestock farming [13,77,78], there is an urgent need to elucidate potential transmission routes. *S. aureus*/MRSA is not only transmitted via person-to-person contact, but can also spread among and between domestic animals, livestock and people via direct contact or inhalation of contaminated air and dust in stables [79,80]. Moreover, pest species, including rats, are a potential source of MRSA [18,19,21]. Taking the “One Health” concept into consideration, measures to control the spread of *S. aureus,* and in particular MRSA, must take these different reservoirs and transmission routes into account. 

Rats actively explore a number of different habitats, and therefore have a unique opportunity to encounter a variety of human and livestock pathogens [16,81,82,83]. Indeed, there is growing evidence that rats play a role in the spread and persistence of MRSA on pig farms [18,21]. Their ability to cover considerable distances allows rats to spread pathogens from one herd to another [84,85]. Besides farm animals, rats might also transfer livestock-derived *S. aureus* from farms to domestic animals or pets, and ultimately to humans through contact with these animals or their excrements. In addition, they might also serve as a ‘mixing vessel’, facilitating the transfer of genetic elements encoding resistance genes either between different *S. aureus* strains or between *S. aureus* and other bacterial species [19]. Thus, strategies to control MRSA on farms should take pest control into account. 

Rats are not only a reservoir for LA-MRSA but also HA-MRSA. In our study, we observed a single CC30-MRSA-positive rat caught within a city. Similarly, Himsworth et al. reported an MRSA-colonization rate of 3.5% among tested urban rats, mostly involving human MRSA common in that area, such as USA300, but also CC398-MRSA [19]. While the risk of rat-to-human MRSA transmission outside the farm setting is very low, it could become relevant in impoverished, inner-city neighborhoods, where socio-economic factors might promote rat infestations and hence rat-to-human contact [19,81,86]. Moreover, the occupational risk of encountering MRSA-colonized rats is quite considerable in case of sewage workers or workers on landfill sites.

In addition to the known infection risk due to colonized free-living wild rats, we report here that captive wild rats may also be a source of MRSA. To avoid transmission and subsequent infection of caretakers, strict hygiene measures, including the use of gloves and face masks, as well as proper disinfection of cages should be implemented. 

The observed elimination of human-specific virulence genes, i.e., IEC-carried immune evasion genes as well as SAg genes, in rat-derived *S. aureus* isolates raises the question, whether they are capable of colonizing and infecting humans. We propose that the potential to colonize and/or infect humans depends on a lineage-specific repertoire of host-specific or broadly-reactive adhesins, immune evasion as well as virulence factors. On the one hand, human colonization and infection with CC49 has only rarely been reported [44,56,87], suggesting a reduced fitness and virulence of these strains in humans. In line with this, caretakers in animal breeding facilities were not colonized with the endogenous rat- or mouse-adapted *S. aureus* strains (personal communication, S. Holtfreter) [45]. 

On the other hand, zoonotic CC398-MRSA are commonly encountered among farm workers or other individuals with professional contact with livestock, thus promoting the spread of CC398-MRSA in the general population and introducing this lineage in hospitals and other healthcare facilities. A recent study demonstrated that these strains are able to cause all types of infections attributed to *S. aureus*; nevertheless, the disease-burden of LA-MRSA seems to be lower compared to other MRSA lineages [78]. The factors driving the spread of this zoonotic lineage in the human population are not understood yet. Interestingly, the livestock-associated CC398 originated from a human CC398-MSSA. Upon host switch, it eliminated the IEC-carrying Sa3int phages and acquired methicillin resistance [88]. Conversely, one could assume that a re-acquisition of these phages promotes the spread of zoonotic CC398-MRSA in the human population. However, a recent study demonstrated that the acquisition of IEC-encoding Sa3int phages is no major driver for the re-adaptation of CC398-MRSA to the human host [89]. 

## 4. Conclusions

*S. aureus*, particularly MRSA, has a major impact not only on human health, but also on farm and companion animals as well as wildlife. Rats are key players in the ecology of MRSA on farms and may also transmit human MRSA. Overall, our data show that rats harbor a natural *S. aureus* population, consisting mainly of CC49- and CC130-MSSA. However, contact with humans or livestock poses a colonization pressure on rats, reflected in the uptake and long-term carriage of *S. aureus* isolates of human or livestock origin. Laboratory rats represent a special case, because they lack the lineages CC49 and CC130 and instead carry lineages that are likely human-derived. Upon transfer to rats, *S. aureus* strains seem to adapt to their new host by removing futile genetic elements carrying human-specific immune evasion genes or SAg genes. Future studies should further investigate *S. aureus* ecology in different settings, e.g., farms versus inner-city environments, as well as among different rat species, i.e. *Rattus rattus* versus *Rattus norvegicus*, and should consider sampling all possible hosts including wild animals (particularly rats), livestock, pets and humans as well as the abiotic environment (e.g., dust samples), to gain a better understanding of transmission and maintenance dynamics. In light of the “One Health” concept, such investigational studies could provide the conceptual foundation for developing effective measures to contain these dangerous pathogens in the future.

## 5. Materials and Methods

### 5.1. Study Design and Ethics Statements 

The subjects of the study were 447 rats (*Rattus norvegicus,* N = 381*; Rattus rattus,* N = 66), mostly from Germany (N = 418), but also from Czech Republic (N = 29), which were either free-living wild rats (N = 145), captive wild rats (N = 188) or laboratory rats (N = 114).

The category “free-living wild rats” comprises rats that were collected in the wild between 2009 and 2017 using live, electric as well as snap traps, in addition to bait poisons, at different locations in Mecklenburg-Western Pomerania (N = 18), North Rhine-Westphalia (N = 81), and Baden-Württemberg (N = 17) [90] as well as in the Moravian-Silesian Region in the Czech Republic (N = 29) (Figure 1). Details on the geographical origin, habitat, and capture method of these free-living wild rats as well as on their characteristics and nasal *S. aureus* colonization are described in Tables S1 and S2, respectively. 

The category “captive wild rats” comprises offspring of wild rats that were bred in a laboratory setting or a large enclosure for several years or even decades. Details on the geographical origin, source, original habitat, housing conditions and hygiene measures in these animal facilities, as well as on the rat characteristics and nasal *S. aureus* colonization are described in Tables S3 and S4, respectively. The category “laboratory rats” comprises domesticated rats raised for animal experiments or as feeder animals (Tables S3 and S5). 

Samples were collected using approved methods, according to relevant legislation and by permission of the responsible State authorities whenever necessary (Supplementary Tables S1 and S3). Wild rats were captured by professional pest control operators, and hence no capture permit was required. The only exception were wild rats from NRW (NRW_2; Supplementary Table S1), which were captured using live traps following approval by the North Rhine-Westphalia State Agency for Nature, Environment and Consumer Protection, No. 84-02.04.2015.A279 (03.11.2015).

Captive wild rats were held in animal facilities in BB, BE, and NRW based on holding permits provided by the responsible Veterinary or State Office at the indicated date (BB: Veterinary Office of the district of Potsdam-Mittelmark (23.06.2014); BE: State Office for Health and Social Issues, No. IC 114 -ZH25 (10.04.2014); NRW: Health and Veterinary Office Münster, No. #39.32.7.1 (09.12.2013). An additional animal ethics permit was granted for animals in NRW by the North Rhine-Westphalia State Agency for Nature, Environment and Consumer Protection, No. 84-02.04.2013.A288 (29.11.2013). 

Laboratory rats were held as feeder animals under a general zoo permit in locations in BW (No. DE 08 111 100115; 12.08.2011) and HE (Darmstadt Regional Council, No. V 51.1 – 1.1 – R 25.3 – ZRL – Kronberg, Opel-Zoo; 19.04.2016). Moreover, laboratory rats were held in laboratory settings under a holding permit from the local authorities in MV (Veterinary and Food Inspection Office, district of Vorpommern-Greifswald, No. Dr.Caa.ZSF3936/11/17; 07.11.2017) and NRW (Health and Veterinary Office Münster, No. #39.32.7.1; 09.12.2013). An additional animal ethics permit was granted for animals in NRW by the North Rhine-Westphalia State Agency for Nature, Environment and Consumer Protection, No. 84-02.04.2013.A288 (29.11.2013). 

Dead animals were immediately frozen and stored at −20 °C until dissection. Their noses were aseptically removed from the body and frozen again at −20 °C.

*S. aureus isolates from laboratory rats:* We also included *S. aureus* isolates in this study, which were obtained from laboratory rats by collaborators. The CR strains (N = 38) were provided by Charles River’s Research Animal Diagnostic Services (Wilmington, USA). Strains were submitted by different Charles River facilities, as well as from Charles River customers (pharmaceutical companies, universities, or research institutes). 13 *S. aureus* isolates were obtained during routine health monitoring or from euthanized rats suffering from *S. aureus* infections (e.g., abscesses) at the animal facilities of the German Cancer Research Center (DKFZ, Deutsches Krebsforschungszentrum, Heidelberg, BW, Germany) in Heidelberg, Germany between 1983 and 2014. A single *S. aureus* isolate was obtained from the caecum of *R. norvegicus* during routine health monitoring at the animal facilities of the University of Ulm (Tierforschungszentrum, Ulm, BW, Germany).

*S. aureus isolates from humans*: Fifty CC-matched human *S. aureus* strains were derived from several *S. aureus* colonization studies (T, SH, SHIP) [44,91,92]. The T and SH strains were obtained from healthy blood donors in Northern Germany in 2002 and 2005–2006, respectively. *Spa* types as well as *agr* type, SAg gene patterns and *S. aureus* integrase (Saint) phage groups of these strains were previously reported [91,92]. The SHIP studies (SHIP-2, SHIP-Trend-0) are population-based studies in Western Pomerania and are described in depth elsewhere [44,93]. Human CC49, CC88, and CC130 isolates were derived from strain collections of the Robert Koch-Institute and the Institute for Medical Microbiology and Hygiene, Technical University of Dresden.

### 5.2. Sample Preparation and Screening for S. aureus 

The rat noses were thawed at room temperature for 1 h and then transferred into tubes containing 1 mL BBL™ Phenol Red Mannitol Broth (MSB: 10 g/L pancreatic digest of casein; 75 g/L NaCl; 5 g/L D-mannitol; 18 mg/L phenol red in double-distilled water). The noses were homogenized using 2 rounds of spinning (6000/min, 2 × 20 s; 15 s breaks) in the presence of 1.4 and 2.8 mm Zirconium oxide beads (Bertin Precellys 24 homogenizer, VWR, Darmstadt, Germany). Aliquots of 100 µL of the homogenate were plated onto mannitol salt agar (MSA) plates (Becton, Dickinson and Co., Franklin Lakes, NJ, USA) and incubated for 48 h at 37 °C. The rest of the homogenate was subsequently transferred to a 50 mL tube and cultured aerobically in MSB (total volume 5 mL) for 48 h at 37 °C under agitation for bacterial enrichment. 

Three random samples from MSA plates containing golden-yellow colonies were used for a mixed-colony PCR to detect *S. aureus* presence by testing for gyrase (*gyr*) and nuclease (*nuc*) genes. In brief, bacterial cells were resuspended in reaction mix (25 μL) containing 1× GoTaq^®^ Flexi buffer, 100 μM deoxynucleoside triphosphates (dATP, dCTP, dGTP, and dTTP; buffer and nucleotides from Thermo Fisher Scientific Baltics UAB, Vilnius, Lithuania), 5 mM MgCl_2_, 320 nmol/L of each primer, 1.0 U GoTaq^®^ G2 Flexi DNA polymerase (Promega, Mannheim, Germany) in DNase- and RNase-free water. PCRs were performed as follows: an initial denaturation step at 95 °C for 10 min was followed by either 30 cycles of amplification (16SrRNA, *nuc*: 95 °C for 30 s, 55 °C for 30 s and 72 °C for 60 s) or 35 cycles of amplification (*gyr*: 95 °C for 30 s, 60 °C for 30 s and 72 °C for 60 s), ending with a final extension phase at 72 °C for 7 min. All PCR products were resolved by electrophoresis in 1.5% agarose gels (1× TBE buffer), stained with RedSafe™ (INtRON Biotechnology, Sungnam, Korea) and visualized under UV light (GenoPlex, VWR, Darmstadt, Germany).

A positive result indicating the presence of *S. aureus* was followed by subculturing 3 distinct colony morphotypes on sheep blood agar (Becton, Dickinson and Co, Franklin Lakes, NJ, USA; 37 °C; 24 h). The colonies were further tested using the *S. aureus*-specific latex agglutination test (Prolex^TM^ Staph Xtra Latex Kit, Pro-Lab Diagnostics, Richmond Hill, ON, Canada). Finally, *S. aureus* isolates were subjected to colony PCR (*gyr*, *nuc*). DNA isolation was performed on all PCR-positive *S. aureus* isolates using the DNeasy Blood and Tissue Kit (Qiagen, Venlo, the Netherlands) according to the manufacturer’s instructions, but with an addition of 0.1 mg/ mL lysostaphin (Sigma-Aldrich, St. Louis, MI, USA) to the lysis buffer [11]. *S. aureus* isolates were stored as glycerol stocks.

### 5.3. S. aureus Identification 

The colonies obtained were screened for *S. aureus* using an *S. aureus*-specific colony multiplex PCR. The amplification of the *16SrRNA* gene served as quality control (756 base pairs (bp); 16SrRNA_forward primer 5′-AAC TCT GTT ATT AGG GAA GAA CA-3′, 16SrRNA_reverse primer 5′-CCA CCT TCC TCC GGT TTG TCA CC-3′) [94], and the *S. aureus*-specific *gyr* gene (281 bp; Gyr_forward primer 5′-AGT ACA TCG TCG TAT ACT ATA TGG-3′, Gyr_reverse primer 5′-ATC ACG TAA CAG TTC AAG TGT G-3′) as well as the *S. aureus* species specific *nuc* gene (279 bp; Nuc_forward primer 5′-GCG ATT GAT GGT GAT ACG GTT-3′, Nuc_reverse primer 5′-AGC CAA GCC TTG ACG AAC TAA AGC-3′) were used to detect *S. aureus* DNA [11,94]. 

### 5.4. Spa Genotyping and Multilocus Sequence Typing (MLST)

Based on the allelic profile of seven house-keeping genes, the species *S. aureus* can be divided into STs, which again are grouped into CCs [95]. S*pa* typing and MLST were performed as described elsewhere [96,97]. *Spa* typing was performed on all *S. aureus* isolates, and isolates were assigned to CCs based on *spa* typing results [34]. MLST was performed on selected isolates to confirm the *spa* clustering results. Profiles were submitted to the SpaServer and the MLST database (https://pubmlst.org/saureus/).

### 5.5. Virulence Gene Detection Using Multiplex PCR 

More than 30 *S. aureus* genes, including *gyr*, methicillin resistance (*mecA*-*mecD*, *mecU*), *agr* group 1–4 genes, as well as the virulence genes for Panton-Valentine leukocidin (*pvl*), staphylococcal superantigens (*sea*-*see*, *seg*-*seu*, *tst*), and exfoliative toxins (*eta*, *etd*) were examined as described elsewhere [92,98]. *S. aureus* bacteriophage types (Sa1int-Sa7int) and the Sa3int phage-carried IEC genes (*sa3int, sak, chp, scn*) were examined with multiplex PCR according to published protocols [44]. 

### 5.6. MIC of Penicillin Against S. aureus Strains Using the Broth Microdilution Method 

The MIC of penicillin G (Merck KGaA, Darmstadt, Germany) was determined in a total volume of 0.1 mL of Mueller-Hinton broth (MHB: casein acid hydrolysate 17.5 g/L; Beef extract 3 g/L; Starch 1.5 g/L; Merck KGaA, Darmstadt, Germany) containing approximately 5 × 10^5^ colony-forming units (CFU)/mL in microdilution trays with round-bottom wells (CELLSTAR^®^, greiner bio-one) according to the CLSI broth microdilution method [99]. The following control strains were included: *S. aureus* ATCC^®^ 43300 (MRSA) and *S. aureus* ATCC^®^ 29213 (*bla^+^*) as positive controls; *S. aureus* ATCC^®^ 25923 (*bla*^-^) as a negative control. In addition, medium sterility as well as growth controls were included in the assay. The penicillin range tested was 0.001–16 µg/mL, whereas the resistance breakpoint was set to ≥0.25 µg/mL according to CLSI guidelines [99]. The inoculated microdilution trays were sealed and incubated at 37 °C for 24–48 h in an ambient air incubator. The MIC values were determined as the lowest penicillin concentration that completely inhibits growth of the organism in the microdilution wells as detected by the unaided eye. 

### 5.7. Antibiograms and mecA-D PCR 

The strains were grown on chromID^®^ MRSA SMART Agar (BioMérieux, Nürtingen, Germany). Susceptibility testing was performed by Vitek 2 AST applying AST-P654 card (BioMérieux). Methicillin resistance was verified by Alere™ PBP2a SA Culture Colony Test (Abbott Rapid Diagnostics, Cologne, Germany) and PCR tests addressing the different *mec* genes as described elsewhere for *mecA*, *mecC* and *mecD* [100,101]. For *mecB* detection, the primer pair mecB-f (5′- GAT GTA CTG TTG CTT CTC TTA A-3′) and mecB-r (5′-CAG AGG GAA AAT ACT AGA C-3′) was designed and tested using *S. aureus* UKM4229 [102] as reference strain.

### 5.8. Coagulation Assay 

Rat CC88 isolates (N = 5) and human CC88 isolates (N = 5), as well as rat CC49 isolates (N = 5) and human CC49 isolates (N = 4) were grown for 7 h at 37 °C and 200 rpm in tryptic soy broth (TSB) to reach the early stationary phase. The OD of the TSB cultures was adjusted to 4. Seventy microliters of the adjusted bacterial cultures (approx. 2.0 × 10^9^ CFU/mL) were mixed in 10 mL glass tubes with 500 µL of rat heparinized plasma from Sprague Dawley rats (Equitech-Bio, Kerrville, TX, USA) and incubated at 37°C without agitation. The coagulation state was visually examined in a blinded fashion after 0.5 h, 1 h, 2 h, 3 h, 4 h, 5 h, 19 h and 24 h using a modified coagulation score [103]. No coagulation was reported as 0, cloudiness of plasma as 1, small coagulation flakes as 2, a medium sized clot as 3, a large clot as 4 and complete coagulation (tube can be inverted) as 5 [45].

### 5.9. Statistical Analyses 

Categorical data were assessed by using Pearson’s Chi-squared test. *p* values of ≤0.05 were considered statistically significant. 

Differences in the coagulation potential between the groups (rat CC49 vs. human CC49; rat CC88 vs. human CC88; human CC49 vs. human CC88, rat CC49 vs. rat CC88) were statistically evaluated per time point by a Kruskal-Wallis test with Dunn’s multiple comparisons test. 

## Figures and Tables

**Table 1:** Prevalence of *Staphylococcus aureus* in free-living wild rats, captive wild rats and laboratory rats.

Category ^1^	Country	State ^2^	No. (Rats)	*S. aureus*^+^ (%)	MRSA (%) ^3^	Pen^R^ (%) ^4^
**Free-living wild**	GER	BW	17	1 (5.9)	1 (5.9)	1 (100.0)
	GER	MV	18	4 (22.2)	0 (0.0)	1 (25.0)
	GER	NRW_1	49	4 (8.2)	1 (2.0)	3 (75.0)
		NRW_2	32	10 (31.3)	0 (0.0)	9 (90.0)
	CZE	MSR	29	18 (62.1)	0 (0.0)	1 (5.6)
		**Total**	**145**	**37 (25.5)**	**2 (1.4)**	**15 (40.5)**
**Captive** **wild**	GER	BB	72	14 (19.4)	5 (6.9)	5 (35.7)
	GER	BE	35	27 (77.1)	1 (2.9)	25 (92.6)
	GER	NRW	81	0 (0.0)	0 (0.0)	0 (0.0)
		**Total**	**188**	**41 (21.8)**	**6 (3.2)**	**30 (73.2)**
**Laboratory**	GER	BW	20	0 (0.0)	0 (0.0)	0 (0.0)
	GER	HE	40	7 (17.5)	0 (0.0)	5 (71.4)
	GER	MV	21	7 (33.3)	0 (0.0)	7 (100.0)
	GER	NRW	33	0 (0.0)	0 (0.0)	0 (0.0)
		**Total**	**114**	**14 (12.3)**	**0 (0.0)**	**12 (85.7)**

^1^ Free-living wild rats: rats that were collected in the wild between 2009 and 2017 using live, electric as well as snap traps, in addition to bait poisons; captive wild rats: offspring of wild rats that were bred in a laboratory setting or a large enclosure for several years or even decades; laboratory rats: domesticated rats raised for animal experiments or as feeder animals. ^2^ For captive wild rats, the state where the animal husbandry is located is provided. ^3^ % relative to the total number of rats tested. ^4^ Determined based on the MIC of penicillin against *S. aureus* strains using the broth microdilution method; resistance breakpoint was set to ≥ 0.25 µg/mL. Abbreviations: BB, Brandenburg; BE, Berlin; BW, Baden-Württemberg; CZE, Czech Republic; GER, Germany; HE, Hesse; MIC, minimum inhibitory concentration; MRSA, methicillin-resistant *S. aureus*; MSR, Moravian-Silesian Region; MV, Mecklenburg-Western Pomerania; No., number; NRW, North Rhine-Westphalia (NRW_1; NRW_2, please refer to supplementary Table S2); Pen^R^, penicillin resistance; %, percentage; **^+^,** positive.

**Table 2:** MRSA isolates from free-living and captive wild rats.

Category ^1^	Strain ID	Habitat ^2^	Country ^3^	State ^3^	Strain	Species	Year	*spa* Type	CC	*mec* Genes ^4^	MRSA agar	Cef^R^	Oxa-MIC (µg/mL)	Interpretation
**Free-living wild**	KS/17/175	town	GER	NRW_1	NA	*Rattus norvegicus*	2016	t685	CC30	*mecA*	+	+	Oxa ≤ 0.25	MRSA ^5^
	KS/17/378	zoo, pest animal	GER	BW	NA	*R. norvegicus*	2012	t843	CC130	*mecC*	+	+	Oxa ≤ 0.25	MRSA ^5^
**Captive wild**	KS/17/19	livestock farm	GER	BB	Neufels	*R. rattus*	2016	t011	CC398	*mecA*	+	+	Oxa ≥ 4	MRSA
	KS/17/20	livestock farm	GER	BB	Neufels	*R. rattus*	2016	t011	CC398	*mecA*	+	+	Oxa ≥ 4	MRSA
	KS/17/21	livestock farm	GER	BB	Neufels	*R. rattus*	2016	t011	CC398	*mecA*	+	+	Oxa ≥ 4	MRSA
	KS/17/22	livestock farm	GER	BB	Neufels	*R. rattus*	2016	t011	CC398	*mecA*	+	+	Oxa ≥ 4	MRSA
	KS/17/46	livestock farm	GER	BB	Neufels	*R. rattus*	2016	t011	CC398	*mecA*	+	+	Oxa ≥ 4	MRSA
	KS/17/390	livestock farm	GER	BE	Neufels	*R. rattus*	2017	t843	CC130	*mecC*	+	+	Oxa ≤ 0.25	MRSA^5^

^1^ For a definition of the different rat categories, please refer to the legend of Table 1. ^2^ For captive wild rats, the habitat of the captured ancestral rats is reported. ^3^ For captive wild rats, the location of the husbandry rather than the capture location is reported. ^4^ PCR for *mecA*, *mecB*, *mecC* and *mecD*. ^5^ Probably heterogenous expression of the PBP2a protein. Abbreviations: BB, Brandenburg; BE, Berlin; BW, Baden-Württemberg; CC, clonal complex; Cef^R^, cefoxitin resistance; GER, Germany; *mec*, methicillin resistance coding gene; MRSA, methicillin-resistant *S. aureus*; NA, not applicable; NRW, North Rhine-Westphalia; Oxa-MIC, oxacillin minimum inhibitory concentration.

**Table 3:** Prevalence of phage-carried IEC genes and MGE-carried SAg genes in rat and matched human isolates ^1^.

		Rat			Human			
		No.	Total	%	No.	Total	%	*p* Value ^2^
**Phage-carried IEC genes**	CC7	6	6	100.0	10	10	100.0	n.s.
	CC8	0	10	0.0	9	10	90.0	*p* < 0.001
	CC49	0	14	0.0	3	4	75.0	*p* < 0.001
	CC88	3	9	33.3	10	10	100.0	*p* < 0.01
	CC130	0	28	0.0	0	9	0.0	n.s.
	CC398	0	15	0.0	5	7	71.4	*p* < 0.001
**MGE-carried SAg genes**	CC7	3	6	50.0	9	10	90.0	n.s.
	CC8	0	10	0.0	7	10	70.0	*p* < 0.01
	CC49	0	14	0.0	0	4	0.0	n.s.
	CC88	0	9	0.0	5	10	50.0	*p* < 0.05
	CC130	0	28	0.0	0	9	0.0	n.s.
	CC398	0	15	0.0	0	7	0.0	n.s.

^1^ Only CCs with N ≥ 5 were considered. To avoid a bias due to highly prevalent clones, a maximum number of five genotypically identical isolates from the same area and year were included. ^2^ Chi-squared test. Abbreviations: IEC, immune evasion cluster; MGE, mobile genetic elements; No., number; n.s., non-significant; SAg, superantigen; %, percentage.

## Supplementary Materials

The following are available online at https://www.mdpi.com/2072-6651/12/2/80/s1. Table S1: Description of the geographical origin, habitat and capture method of free-living wild rats (N = 145). Table S2: Overview of the wild rats included in this study, their origin, characteristics and nasal *S. aureus* colonization. Table S3: Description of the geographical origin, original habitat, housing conditions and hygiene measures in husbandries of captive wild rats (N = 188) as well as laboratory rats (N = 114) from Germany. Table S4: Overview of the captive wild rats included in this study, their origin, characteristics and nasal *S. aureus* colonization. Table S5: Overview of the laboratory rats included in this study, their origin, characteristics and nasal *S. aureus* colonization. Table S6: Genotype, virulence genes, phage patterns, and penicillin resistance of *S. aureus* isolates from wild rats. Table S7: Genotype, virulence genes, phage patterns, and penicillin resistance of *S. aureus* isolates from captive wild rats. Table S8: Genotype, virulence genes, phage patterns, and penicillin resistance of *S. aureus* isolates from laboratory rats. Table S9: Genotype, virulence genes, and phage patterns of selected CC-matched human *S. aureus* isolates.
